# Early re-examination of chest CT may be unnecessary when patients with psittaci pneumonia at low and intermediate risk on Pneumonia Severity Index present with response to initial treatment

**DOI:** 10.3389/fmed.2025.1574706

**Published:** 2025-06-11

**Authors:** Ming Chen, Shu Ying Zeng, Sheng Jia Lu, Miao Shi, Qing Qian Zhang

**Affiliations:** ^1^Department of Respiratory and Critical Care Medicine, Tongde Hospital of Zhejiang Province, Hangzhou, China; ^2^Clinical Medical College of Integrated Chinese and Western Medicine, Zhejiang Chinese Medicine University, Hangzhou, China; ^3^Infectious Diseases, Tongde Hospital of Zhejiang Province, Hangzhou, China; ^4^Gynecology, Tongde Hospital of Zhejiang Province, Hangzhou, China; ^5^Department of Oncology, Tongde Hospital of Zhejiang Province, Hangzhou, China

**Keywords:** parrot fever, Chlamydia pneumonia, intermediate pneumonia severity index, chest computed tomography, inflammatory markers

## Abstract

**Background:**

Chlamydia parrot pneumonia (CPS) is a rare community-acquired pneumonia (CAP) caused by Chlamydia parrot infection. With the development of metagenomic second-generation sequencing technology (mNGS), its diagnostic rate has improved in recent years. However, there are few clinical studies on Chlamydia parrot pneumonia, especially for patients with low and intermediate pneumonia severity index (PSI), and the necessity of early review of chest computed tomography (CT) is not clear. This study aimed to explore the clinical significance of early review of chest CT in patients with low and intermediate risk of Chlamydia parrot pneumonia with PSI after initial treatment was effective.

**Methods:**

Retrospective analysis of 8 patients with *Chlamydia psittaci* pneumonia diagnosed by metagenomic next-generation sequencing (mNGS) admitted to Zhejiang Provincial Tongde Hospital from January 2020 to December 2022 (PSI score ≤ 130 points). All patients had improved clinical symptoms and inflammatory markers after receiving antibiotic treatment, and chest CT was reexamined within 5-12 days. Evaluate the correlation between imaging changes before and after treatment and clinical symptoms and inflammatory indicators (CRP, PCT, WBC, etc.).

**Results:**

After treatment, the patient’s body temperature, CRP, PCT and other indicators decreased significantly (*P* < 0.05). However, early CT reexamination showed that imaging progressed in 5 cases (62.5%), was stable in 2 cases (25%), and only 1 case (12.5%) showed partial improvement (*P* > 0.05). Nevertheless, none of the patients had a deterioration in their condition later on and finally achieved imaging recovery. Clinicians did not adjust the treatment plan when imaging progressed, and only 1 case was given glucocorticoid additionally.

**Conclusion:**

For patients with low- intermediate risk *Chlamydia psittaci* pneumonia with low PSI, if clinical symptoms and inflammatory markers improve, early reexamination of chest CT may have no additional clinical value and does not affect treatment decisions. Therefore, it is not recommended to routinely perform early CT re-examination for such patients to reduce unnecessary consumption of medical resources. Larger sample studies are needed in the future for further verification.

**Clinical trial registration:**

https://www.medicalresearch.org.cn/login, identifier MR-33-25003507.

## 1 Introduction

*Chlamydia psittaci* pneumonia is a zoonotic infectious disease caused by *Chlamydia psittaci* infection, which may be asymptomatic in mild cases and lead to severe pneumonia and multiple organ failure, and even death (1, 2). It is mainly transmitted to humans through contact with infected birds or inhalation of bacterial-containing aerosols or dust such as secretions and excretions of infected birds, with birds (e.g., parrots and poultry) as the main hosts (3). In recent years, with the gradual development and maturity of metagenomic next-generation sequencing (mNGS) and its ability to rapidly and accurately detect a wide range of pathogens, more and more *Chlamydia psittaci* pneumonia has been clearly diagnosed (4). *Chlamydia psittaci* pneumonia accounts for community acquired pneumonia (CAP) ranging from 0.5 to 15%, with an average of 1%, and is a relatively rare cause of CAP (5). Therefore, clinicians are much less familiar with it than with common pathogens, and the clinical studies published on *Chlamydia psittaci* pneumonia are mostly descriptive, with less attention paid to the interrelationships between clinical symptoms, inflammatory markers, and imaging in the diagnosis and management of the disease. For most clinicians, *Chlamydia psittaci* is a relatively unfamiliar pathogen, so the assessment of changes in its condition may be positive and comprehensive to ensure that mistakes do not occur during diagnosis and treatment. For example, when symptoms are clinically assessed to be improving and inflammatory markers are decreasing, chest CT may also be repeated simultaneously to further evaluate and confirm whether the treatment regimen is effective. The necessity of chest CT re-examination remains uncertain for patients with low-to-intermediate risk Pneumonia Severity Index (PSI) scores. In addition, it is also possible to face an incomplete match between clinical improvement but no significant absorption or even progressive imaging, and this situation will affect the adjustment of the subsequent treatment plan. At present, there is limited data available to summarize the above issues, therefore, this study intends to retrospectively analyze the clinical data and pre- and post-treatment images of *Chlamydia psittaci* pneumonia cases with low and intermediate-risk PSI who were admitted to our hospital with the aim of clarifying whether early re-examination of chest CT is clinically significant, and also to better understand the clinical characteristics of *Chlamydia psittaci* pneumonia patients, so as to facilitate the implementation of a better management strategy.

## 2 Materials and methods

### 2.1 Research objects

Collect patients diagnosed with *Chlamydia psittaci* pneumonia by mNGS who were hospitalized in Zhejiang Provincial Tongde Hospital from January 1, 2020 to December 30, 2022.

### 2.2 Collect data

#### 2.2.1 General information

Demographic characteristics such as age, gender, occupational history and history of contact with poultry, symptoms, vital signs (body temperature, heart rate, respiratory rate); treatment plan.

#### 2.2.2 Laboratory examinations and imaging examinations

After the patient was admitted to the hospital, blood samples were collected in accordance with the standard venous blood collection procedure. Venous blood routine and blood biochemistry were analyzed by a full blood cell analyzer and a blood biochemical analyzer, respectively. Procalcitonin was analyzed by a chemiluminescent immunoanalyzer. The patient’s blood routine [white blood cells (WBC), neutrophils, hypersensitive C-reactive protein (CRP)]; biochemistry [lactate dehydrogenase (LDH), phosphokinase (CK), alanine aminotransferase (ALT), aspartate aminotransferase (AST)]; and procalcitonin (PCT) were collected. A 128-slice multi-slice spiral computed tomography (CT) was used to perform chest scans on the patient.

#### 2.2.3 mNGS detection: sample collection process and requirements

The sample collection volume requirements were as follows: Bronchoalveolar lavage fluid (3-5 mL) was collected into a special sample tube (with preservation solution) and transported with dry ice within 48 h. For sputum samples, 0.1% DTT (dithiothreitol) was added before nucleic acid extraction, and the sample was placed at room temperature for 30 min for liquefaction. For swab samples, 450 μL PBS was added and shaken for 5 min before nucleic acid extraction. Single D detection process: For sample types other than blood, the PathoXtract^®^ Basic universal pathogen nucleic acid extraction kit (WYXM03211S, Micro Rock Medicine) was used for DNA extraction. DNA was eluted with 50 μL of nuclease-free water. Library construction and sequencing: The KAPA DNA HyperPrep Kit (KK8504, KAPA, Kapa Biosystems, Wilmington, MA, United States) was used to construct the cell-free DNA library. The pooled library was sequenced on an Illumina NextSeq 550Dx sequencer with 75 bp single-end sequencing. Approximately 20 M sequencing reads were obtained for each sample. In each batch of experiments, a negative control (nuclease-free water) and a positive control (Klebsiella pneumoniae, ATCC 700603) were included for routine experiments to ensure quality control of the experimental process.

### 2.3 Diagnostic criteria

All cases were confirmed by finding *Chlamydia psittaci* sequences through metagenomic next-generation sequencing (mNGS) of bronchoalveolar lavage fluid.

### 2.4 Inclusion criteria

(1) Patients with pneumonia assessed by Pneumonia Severity Index (PSI) who scored less than 130 points for low- and intermediate-risk patients (6); (2) Patients who were considered to respond to initial treatment by appropriate antibiotic therapy and had chest CT repeated about 1 week later.

### 2.5 Effective initial treatment

Clinical stability was achieved after treatment and could be considered effective initial treatment. Clinical stability criteria were to meet all 5 of the following criteria: (1) body temperature ≤ 37.8°C; (2) heart rate ≤ 100 beats/min; (3) respiratory rate ≤ 24 breaths/min; (4) systolic blood pressure ≥ 90 mmHg; (5) oxygen saturation ≥ 90% (or partial pressure of arterial oxygen ≥ 60 mmHg under air inhalation) (7, 8), and here we paid more attention to the decrease in body temperature (9). CRP was also considered a marker of response to treatment (10, 11). A decrease in CRP was defined if CRP levels fell into the normal range (< 0.5 mg/dL) or was reduced compared with the peak level before treatment.

### 2.6 Imageology evaluation

We based our analysis on four categories of radiographic changes, described by the international standard nomenclature of the Fleischner Society Glossary of Terms (12). Referring to previous criteria for invasive fungal disease (13, 14) as follows: “imaging progression”: defined as emerging or increasing areas of parenchymal attenuation including consolidation, ground-glass opacity, or pleural effusion; “imaging stabilization”: defined as no significant change from previous; “partial response”: defined as a decrease from previous abnormal findings on chest CT; “complete response”: defined as complete absorption of previous abnormal chest CT images.

### 2.7 Statistical analysis

Use SPSS 19.0 software to analyze data. Measurement data are expressed as mean ± standard deviation (x ± s), and qualitative data are expressed as frequency (percentage). For measurement data that conform to a normal distribution, paired *t*-tests are used for comparison before and after treatment. For comparison between groups of measurement data that do not conform to a normal distribution, Mann-Whitney *U*-tests are used. Chi-square tests are used for comparison between groups of dichotomous count data. Rank-sum tests are used for grade data. A *P* < 0.05 is considered statistically significant.

## 3 Results

### 3.1 Demographic data characteristics

We collected a total of 15 patients diagnosed with *Chlamydia psittaci* pneumonia by mNGS. Among them, a total of 8 patients met the inclusion criteria. Their baseline demographic and clinical characteristics are shown in Table 1. Demographic characteristics: Among the 8 patients, there were 2 males and 6 females; aged 48-73 years old, with an average age of 61 years old; 5 patients had a definite history of contact with birds or poultry, mainly raising parrots; some patients had underlying diseases such as hypertension, but there were no diseases that cause immunodeficiency. Clinical manifestations and laboratory characteristics: The time from onset to admission was 2.5 days; all patients had fever during the course of the disease, with an average body temperature of 39.4°C. There were 7 cases with the highest body temperature at 39°C or above. Other symptoms included fever (100%, 8/8), cough (37.5%, 3/8), fatigue (12.5%, 1/8), and headache (37.5%, 3/8). The average PSI score on admission was 61.

**TABLE 1:** Demographic characteristics of 8 patients.

Serial number	Age	Gender	Tmax	Cough	Other symptoms	Underlying disease	Contact history	PSI
1	48	Female	39.6	( + )	Headache and chest pain	Good health	No	48
2	54	Female	40	(−)	(−)	Good health	No	69
3	65	Male	39.3	( + )	Muscle soreness and weakness	Hypertension, asthma.	No	65
4	64	Female	40.2	(−)	(−)	Hypertension, osteoporosis	Yes	69
5	72	Female	39.6	(−)	Headache	Hypertension	Yes	72
6	73	Male	39	(−)	Headache	Hypertension	Yes	73
7	56	Female	40.4	(−)	(−)	Good health	Yes	46
8	57	Female	37.6	( + )	(−)	Hypertension	Yes	47

### 3.2 Serological index characteristics

The distribution of the patients’ first serological results is shown in Table 2. The total number of WBC in patients with *Chlamydia psittaci* pneumonia included in the study is almost all within the normal range, with only one case having WBC exceeding 10 × 109/L. The proportion of neutrophils is all increased, but the absolute value of neutrophils is not all increased. CRP is increased in all cases. When admitted to the hospital, eight patients underwent PCT detection, and one of them was higher than 0.5 μg/L. Lactate dehydrogenase and creatine kinase are almost normal. Some patients show a mild increase in AST/ALT (37.5%, 3/8). In five patients, the blood sodium is slightly decreased (62.5%, 5/8).

**TABLE 2:** Results of first serological indicators of 8 patients.

Serial number	WBC	N%	ANC	CRP	PCT	Na	LDH	ALT	AST	CK
1	7.1	79.8	5.6	81.6	0.074	134.6	162	23	34	54
2	6.1	81.8	5.0	171.3	0.25	131.5	230	64	59	79
3	13.5	84.9	11.5	264.3	1.69	134.9	237	20	38	215
4	7.5	79.2	5.9	79.3	0.19	133.3	221	75	41	153
5	9.4	77.9	7.3	85.8	0.16	137.8	161	22	27	23
6	8.4	86.5	7.3	131.2	0.22	137.6	229	55	72	145
7	6.6	79.7	5.3	105.1	0.33	135.3	202	28	29	95
8	9	70.7	6.4	22	0.06	141	145	14	18	86

(Blood indicators normal range: Blood Na 137-147 mmol/L; WBC 3.5-9.5*109/L; N% 40-70%; CRP 0-6 mg/L; PCT < 0.065 μg/L; LDH 120-250 μ/L; AST 7-40 μ/L; ALT 15-35 μ/L; CK 40-200 μ/L).

### 3.3 Treatment plan and imaging description before treatment

The description of the results of the patients’ first chest CT is shown in Table 3. The chest CT results show that there are 3 cases (37.5%) of pulmonary consolidation, 3 cases (37.5%) of consolidation with patchy shadows, 1 case (12.5%) of consolidation with a small amount of pleural effusion, and 1 case (12.5%) of simple patchy shadows. The treatment plans for patients are shown in Table 3. Four patients use doxycycline as an antibiotic, one patient uses moxifloxacin, and three patients use doxycycline combined with moxifloxacin for treatment.

**TABLE 3:** Treatment regimens and imaging features before treatment of 8 patients.

Serial number	Imaging features	Treatment plan
1	Solid change	Doxycycline
2	Solid change, a small amount of pleural effusion	Moxifloxacin
3	Solid change	Moxifloxacin + Doxycycline
4	Solid change, patchy shadow	Moxifloxacin + Doxycycline
5	Solid change, patchy shadow	Doxycycline
6	Solid change, patchy shadow	Doxycycline
7	Solid change	Moxifloxacin + Doxycycline
8	patchy shadow	Doxycycline

### 3.4 Evaluation of differences before and after treatment

Paired *t*-test was used to statistically analyze WBC, CRP, PCT, and Tmax before and after treatment. The results showed that serum indexes and maximum body temperature after treatment were significantly lower than those before treatment, and the difference was statistically significant (*P* < 0.05) (see Table 4).

**TABLE 4:** Comparison of differences before and after treatment.

Laboratory indicators		X ± S	*t*	*P*
WBC	Before treatment	8.45 ± 2.34	5.374	<0.01
	After treatment	5.375 ± 1.65		
CRP	Before treatment	117.57 ± 73.34	5.17	<0.01
	After treatment	37.75 ± 32.36		
PCT	Before treatment	0.37 ± 0.53	1.72	<0.01
	After treatment	0.06 ± 0.03		
Tmax	Before treatment	39.46 ± 0.88	11.39	<0.01
	After treatment	36.68 ± 0.39		

### 3.5 Imaging changes before and after treatment

**FIGURE 1:**
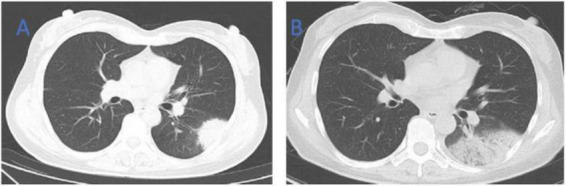
Chest CT of patient 1. **(A)** Before treatment on September 7, 2022; **(B)** the follow-up imaging 1 week after treatment showed progression compared to the pre-treatment scans.

**FIGURE 2:**
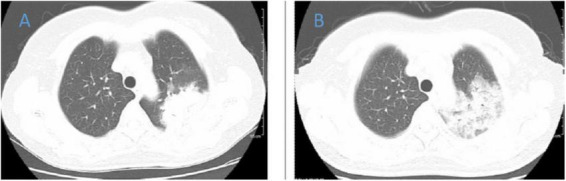
Chest CT of patient 2. **(A)** Before treatment on 2020.8.29; **(B)** the follow-up imaging 1 week after treatment showed progression compared to the pre-treatment scans.

**FIGURE 3:**
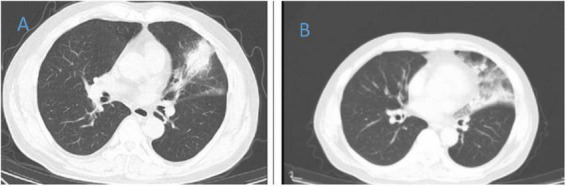
Chest CT of patient 3. **(A)** Before treatment on 2021.12.11; **(B)** the follow-up imaging 1 week after treatment showed progression compared to the pre-treatment scans.

**FIGURE 4:**
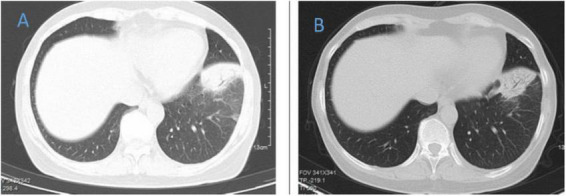
Chest CT of patient 4. **(A)** Before treatment on 2021.10.26. **(B)** The follow-up imaging at 1 week post-treatment showed stable findings compared to the pre-treatment scans.

**FIGURE 5:**
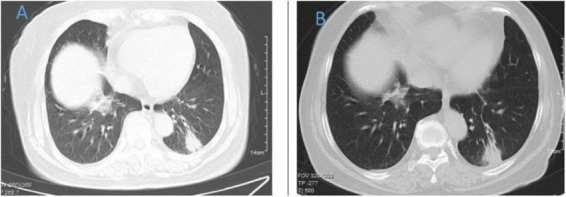
Chest CT of patient 5. **(A)** Before treatment on 2021.10.19. **(B)** The follow-up imaging at 1 week post-treatment showed stable findings compared to the pre-treatment scans.

**FIGURE 6:**
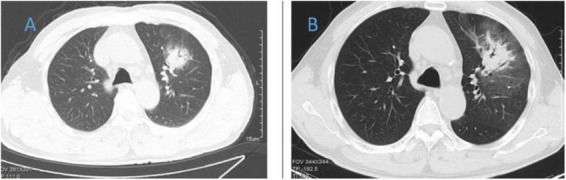
Chest CT of patient 6. **(A)** Before treatment on 2021.10.19. **(B)** The follow-up imaging 1 week after treatment showed progression compared to the pre-treatment scans.

**FIGURE 7:**
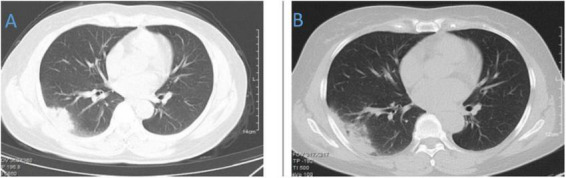
Chest CT of patient 7. **(A)** Before treatment on 2021.4.19. **(B)** The follow-up imaging 1 week after treatment showed progression compared to the pre-treatment scans.

**FIGURE 8:**
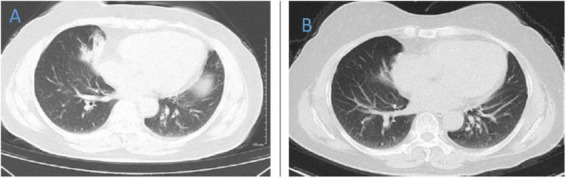
Chest CT of patient 8. **(A)** Before treatment on 2022.1.10. **(B)** The follow-up imaging 1 week after treatment showed improvement compared to the pre-treatment scans.

Eight patients included in the retrospective analysis all underwent chest CT reexamination after the initial treatment was effective. The reexamination time was 5-12 days after the first CT. Compared with the imaging examination before admission, only 1 case showed reduced exudation, and the imaging suggested partial improvement; 5 cases showed an increased range of exudation, and a small number had pleural effusion, and the imaging suggested progression; 2 cases showed that the consolidation and exudation range were similar to before, and the imaging suggested stability. There was no statistically significant difference (*P* > 0.05). For patients with imaging suggesting progression, there was no major change in the decision of clinicians, and the original treatment plan was continued. During the continuous treatment with the original plan, there was no instability of clinical symptoms or repeated inflammatory markers. In the subsequent chest CT reexamination, it was also indicated that the pulmonary exudative lesions and pleural effusion were gradually absorbed, reaching imaging cure (see Table 5 and Figures 1–8).

**TABLE 5:** Comparative analysis of imaging changes before and after treatment.

Serial number	Imaging features after treatment (5-12 days)	Improvement degree	*P*
1	Solid change, a small amount of pleural effusion	Progress	0.69
2	Solid changes, exudation is increased compared with before.	Progress	
3	Solid change, a small amount of pleural effusion	Progress	
4	patchy shadow	Partial improvement	
5	Solid changes and patchy shadows	stable	
6	Solid change, a small amount of pleural effusion	progress	
7	Solid changes, Exudation increases compared with before.	Progress	
8	Patchy shadow	Stable	

### 3.6 Follow-up

Eight patients all showed clinical improvement after the treatment plan was adjusted to an appropriate one. Specifically, the body temperature gradually returned to normal (48-72 h), and blood inflammation indicators decreased. After treatment, none of the cases developed into severe pneumonia (see Table 6).

**TABLE 6:** Follow-up of disease changes after patient discharge.

	Treatment day 5-12 d	Follow-up after discharge
Progression of disease	5	0
Stable response	2	0
Partial response	1	0
Complete response	0	8

## 4 Discussion

For most patients with CAP, clinical symptoms should improve within 72 h when initial treatment is effective, and relevant guidelines and literature also mention that imaging improvement lags behind clinical symptoms (15–17). The above views are proposed based on the common pathogens causing community-acquired pneumonia in clinical practice at present, but community-acquired pneumonia caused by *Chlamydia psittaci* is relatively rare, and has only been relatively reported in recent years. Knowledge of this issue is much less familiar than other common pathogens and are case reports, descriptive studies, and the relationship between improvement in clinical symptoms and imaging in *Chlamydia psittaci* pneumonia has not been reported, and it is unclear whether *Chlamydia psittaci* pneumonia also shows the same pattern. To the best of our knowledge, the present study is the first article focusing on clinical symptom improvement and imaging changes in *Chlamydia psittaci* pneumonia with a (PSI) in the low and intermediate range.

Typical clinical symptoms of *Chlamydia psittaci* pneumonia are mainly high fever, cough, dyspnea, fatigue, chills, headache, and muscle soreness. The patients with *Chlamydia psittaci* pneumonia included in our study all had fever during the course of the disease, and high fever above 39°C was more common. Extra-pulmonary manifestations were mainly fatigue and headache, and digestive system symptoms were not obvious. The results of the first laboratory examination of patients suggest that the total number of white blood cells is not high, CRP is increased, PCT is basically not increased, LDH and CK are basically normal, and some patients show a slight increase in ALT and AST, and a slight decrease in blood sodium. Psittacosis often involves lung tissue. Although chest CT examination cannot directly diagnose, it can quickly clarify the infection site and lesion range, providing a basis for clinical diagnosis and treatment. In short, the diagnosis of *Chlamydia psittaci* pneumonia needs to be comprehensively determined based on the patient’s clinical manifestations, contact history and etiological results.

The “Chinese Expert Consensus on Diagnosis and Treatment of Psittacosis” indicates that tetracyclines, including doxycycline and omadacycline, are used as first-line antibiotics for *Chlamydia psittaci* pneumonia; macrolide antibiotics are used for second-line treatment; other treatments can choose quinolone antibiotics. The clinical symptoms of the patients in this study improved rapidly after being treated with empirical and effective antibiotics after a definite diagnosis was adjusted, and inflammatory markers decreased. None of the cases developed into severe pneumonia or died. This is not completely consistent with the reports of other researchers (18, 19). We analyzed that the reason is that the patients included in this study are relatively mild, all patients with mild to moderate PSI. Other researchers did not use PSI to distinguish for stratified comparison, and some patients with severe conditions were included. On the other hand, it also suggests that if PCT, LDH, CK, ALT, and AST are significantly increased and blood sodium is significantly decreased, it indicates a more severe condition. In order to find out whether it is meaningful to re-examine imaging early in patients with *Chlamydia psittaci* pneumonia who were effectively treated with initial therapy, we included patients who responded to initial treatment and re-examined chest CT during hospitalization. Because it was a retrospective study, and each patient who underwent re-examination of chest CT would differ, it was not possible to accurately define the specific “early stage.” At the same time, in order to reduce the interference of other factors, such as severe immunosuppressive status, we selected patients with low and intermediate PSI scores. Most of the included cases showed no improvement in chest CT, and even a small proportion showed imaging progression compared with chest CT before admission. There was a temporal “separation” between imaging, clinical symptoms and improvement in blood inflammatory markers. In our retrospective study, we showed that as long as the patient’s clinical symptoms continue to improve and the blood inflammatory markers gradually return to baseline, there is no need to pay extra attention. On subsequent follow-up, almost complete resorption of the original lesions and minimal striate and fibrosis after re-examination of chest CT could achieve radiologic cure, so we believe that the improvement in imaging in patients with *Chlamydia psittaci* pneumonia lags behind clinical symptoms and blood inflammatory markers.

Whether the inconsistency between initial treatment improvement and radiographic performance affects the clinician’s subsequent treatment decisions was determined by retrospectively analyzing the treatment regimens of the 8 patients mentioned above, with no change in treatment regimen in 7/8 patients, and the clinician adding a small amount of glucocorticoids in 1 patient. Therefore, we believe that early imaging non-response or even progression compared to initial treatment will not have a great impact on treatment decisions. For another patient taking glucocorticoids, clinicians may consider it helpful in reducing radiographic exudation. In the absence of published treatment guidelines specifically for *Chlamydia psittaci* infection, most of the adjustments to this regimen were based on the experience of clinicians or on regimens for other pathogen infections, such as viruses (literature), which, of course, required more data to support it.

We also communicated with the attending physicians of the patients included in this retrospective study to try to understand what factors prompted the attending physicians to make the decision to perform early re-examination of chest CT when the initial treatment was effective. They believed that compared with pneumonia caused by other pathogens, there was less understanding of Chlamydia psittaci. The support of more imaging would determine the correctness and effectiveness of the previous treatment plan, and would also be more conducive to ensuring the safety of patients.

On the other hand, whether follow-up chest CT needs to be continued after discharge from the hospital with improved condition, our retrospective study showed that the physician in charge would recommend follow-up chest CT, while the patient was also very cooperative in completing the follow-up of chest CT. Chest radiography or chest CT is an important diagnostic tool in patients with suspected lower respiratory tract infection to confirm or exclude the diagnosis of pneumonia. Relevant guidelines consider routine chest imaging is not recommended for patients with significant improvement in clinical symptoms, and chest X-ray or chest CT should be repeated to identify changes in lung lesions only when symptoms or signs persist or worsen (20). However, we believe that follow-up chest CT remains important, and in addition to assessing whether this pneumonia has completely improved or left other forms of disease, confirmation of complete radiological clearance is important to rule out non-infectious potential causes of pneumonia or radiological abnormalities, such as tumors. In addition, when patients with pneumonia present with mild symptoms, such as mild cough, no fever, and even low inflammatory parameters, follow-up chest CT for comparison before and after, may be the most direct or even the only way to confirm whether the pneumonia is cured.

In summary, *Chlamydia psittaci* pneumonia patients with low and moderate PSI also showed imaging improvement lagging behind the clinical symptoms and blood inflammatory indicators. Our results support that for patients who have shown improvement in clinical symptoms and decrease in blood inflammatory markers, early re-examination of chest CT seems to have no additional value in the clinical course of patients and can also lead to unnecessary waste of resources, so we do not recommend early routine re-examination of chest CT for this part of patients.

This study is a retrospective study with small sample size, which has some limitations. Because of the insufficient number of subjects, it is not evaluated whether it is of special significance to re-examine chest CT in patients with *Chlamydia psittaci* pneumonia at high risk by pneumonia severity index (PSI) at an early stage as well as determining the optimal time for subsequent follow-up chest CT, which are questions of significance.

## Data Availability

The original contributions presented in this study are included in this article/supplementary material, further inquiries can be directed to the corresponding authors.

## References

[1] Kong C Zhu J Lu J Xu Z. Clinical characteristics of Chlamydia psittaci pneumonia. *Chin Med J (Engl).* (2021) 134:353–5. 10.1097/CM9.0000000000001313 33410632 PMC7846496

[2] Shi Y Chen J Shi X Hu J Li H Li X A case of chlamydia psittaci caused severe pneumonia and meningitis diagnosed by metagenome next-generation sequencing and clinical analysis: A case report and literature review. *BMC Infect Dis.* (2021) 21:621. 10.1186/s12879-021-06205-5 34193063 PMC8243071

[3] Balsamo G Maxted A Midla J Murphy J Wohrle R Edling T Compendium of measures to control chlamydia psittaci infection among humans (Psittacosis) and pet birds (Avian Chlamydiosis), 2017. *J Avian Med Surg.* (2017) 31:262–82. 10.1647/217-265 28891690

[4] Miao Q Ma Y Wang Q Pan J Zhang Y Jin W Microbiological diagnostic performance of metagenomic next-generation sequencing when applied to clinical practice. *Clin Infect Dis.* (2018) 67:S231–40. 10.1093/cid/ciy693 30423048

[5] Hogerwerf L De Gier B Baan B Van D Hoek W. Chlamydia psittaci (psittacosis) as a cause of community-acquired pneumonia: A systematic review and meta-analysis. *Epidemiol Infect.* (2017) 145:3096–105. 10.1017/S0950268817002060 28946931 PMC9148753

[6] Fine M Auble T Yealy D Hanusa B Weissfeld L Singer D A prediction rule to identify low-risk patients with community-acquired pneumonia. *N Engl J Med.* (1997) 336:243–50. 10.1056/NEJM199701233360402 8995086

[7] Wang D Willis D Yih Y. The pneumonia severity index: Assessment and comparison to popular machine learning classifiers. *Int J Med Inform.* (2022) 163:104778. 10.1016/j.ijmedinf.2022.104778 35487075

[8] Menéndez R Torres A Rodríguez de Castro F Zalacaín R Aspa J Martín Villasclaras JJ Reaching stability in community-acquired pneumonia: The effects of the severity of disease, treatment, and the characteristics of patients. *Clin Infect Dis.* (2004) 39:1783–90. 10.1086/426028 15578400

[9] Mandell L Wunderink R Anzueto A Bartlett J Campbell G Dean N lnfectious diseases society of America/American thoracic society consensus guidelines on the management of community-acquired pneumonia in adults. *Clin lnfect Dis.* (2007) 44:527–72. 10.1086/511159 17278083 PMC7107997

[10] Woodhead M Blasi F Ewig S Garau J Huchon G Ieven M Joint Taskforce of the European respiratory society and European society for clinical microbiology and infectious diseases. guidelines for the management of adult lower respiratory tract infections–full version. *Clin Microbiol Infect.* (2011) 17:E1–59. 10.1111/j.1469-0691.2011.03672.x 21951385 PMC7128977

[11] Ruiz-González A Falguera M Porcel J Martínez-Alonso M Cabezas P Geijo P C-reactive protein for discriminating treatment failure from slow responding pneumonia. *Eur J Intern Med.* (2010) 21:548–52. 10.1016/j.ejim.2010.09.006 21111942

[12] Hansell D Bankier A MacMahon H McLoud T Müller N Remy J. Fleischner Society: Glossary of terms for thoracic imaging. *Radiology.* (2008) 246:697–722. 10.1148/radiol.2462070712 18195376

[13] Segal B Herbrecht R Stevens D Ostrosky-Zeichner L Sobel J Viscoli C Defining responses to therapy and study outcomes in clinical trials of invasive fungal diseases: Mycoses study group and European organization for research and treatment of cancer consensus criteria. *Clin Infect Dis.* (2008) 47:674–83. 10.1086/590566 18637757 PMC2671230

[14] Jung J Hong H Lee S Choi S Kim Y Woo J Immune reconstitution inflammatory syndrome in neutropenic patients with invasive pulmonary aspergillosis. *J Infect.* (2015) 70:659–67. 10.1016/j.jinf.2014.12.020 25597823

[15] British Thoracic Society Standards of Care Committee. BTS guidelines for the management of community acquired pneumonia in adults. *Thorax.* (2001) 56:IV1–64. 10.1136/thorax.56.suppl_4.iv1 11713364 PMC1765992

[16] Bruns A Oosterheert J El Moussaoui R Opmeer B Hoepelman A Prins J. Pneumonia recovery: Discrepancies in perspectives of the radiologist, physician and patient. *J Gen Intern Med.* (2010) 25:203–6. 10.1007/s11606-009-1182-7 19967464 PMC2839328

[17] Bruns A Oosterheert J Prokop M Lammers J Hak E Hoepelman A. Patterns of resolution of chest radiograph abnormalities in adults hospitalized with severe community-acquired pneumonia. *Clin Infect Dis.* (2007) 45:983–91. 10.1086/521893 17879912

[18] Xiao Q Shen W Zou Y Dong S Tan Y Zhang X Sixteen cases of severe pneumonia caused by Chlamydia psittaci in South China investigated via metagenomic next-generation sequencing. *J Med Microbiol.* (2021) 70: 10.1099/jmm.0.001456 34817316

[19] Ni Y Zhong H Gu Y Liu L Zhang Q Wang L Clinical features, treatment, and outcome of psittacosis pneumonia: A multicenter study. *Open Forum Infect Dis.* (2023) 10:ofac518. 10.1093/ofid/ofac518 36817742 PMC9937045

[20] Metlay J Waterer G Long A Anzueto A Brozek J Crothers K Diagnosis and treatment of adults with community-acquired pneumonia. An official clinical practice guideline of the American thoracic society and infectious diseases society of America. *Am J Respir Crit Care Med.* (2019) 200:e45–67. 10.1164/rccm.201908-1581ST 31573350 PMC6812437

